# Effects of acute normovolemic hemodilution and allogeneic blood transfusion on postoperative complications of oral and maxillofacial flap reconstruction: a retrospective study

**DOI:** 10.1186/s12903-024-04302-w

**Published:** 2024-05-24

**Authors:** Wenhao Li, Xueer Li, Yanhong Chen, Yanling Li, Rui Chen, Ziqin Kang, Zhiquan Huang, Yili Zhao

**Affiliations:** 1 Department of Oral and Maxillofacial Surgery, Sun Yat-sen Memorial Hospital, 107th Yanjiang Xi Road, Guangzhou, Guangdong, 510120 China; 2 Department of Maxillofacial Surgery, The First Affiliated Hospital of Guangdong Pharmaceutical University, 19th Nonglinxia Road, Guangzhou, Guangdong, 510080 China; 3 Department of Transfusion Medicine, Sun Yat-sen Memorial Hospital, 107th Yanjiang Xi Road, Guangzhou, Guangdong, 510120 China

**Keywords:** Oral and maxillofacial flap reconstruction, Allogeneic blood transfusion, Acute normovolemic hemodilution, Blood index changes, Postoperative complications

## Abstract

**Objective:**

Patients undergoing oral and maxillofacial flap reconstruction often need blood transfusions due to massive blood loss. With the increasing limitations of allogeneic blood transfusion (ABT), doctors are considering acute normovolemic hemodilution (ANH) because of its advantages. By comparing the differences in the (Δ) blood indices and postoperative complications of patients receiving ABT or ANH during the reconstruction and repair of oral and maxillofacial tumor flaps, this study’s purpose was to provide a reference for the clinical application of ANH.

**Methods:**

The clinical data of 276 patients who underwent oral and maxillofacial flap reconstruction from September 25, 2017, to October 11, 2021, in the Department of Oral and Maxillofacial Surgery, Sun Yat-sen Memorial Hospital, were retrospectively analyzed. According to the intraoperative blood transfusion mode, the patients were divided into two groups: ABT and ANH. The differences in the (Δ) blood indices and the incidence of postoperative complications between the groups were analyzed.

**Results:**

Among the 276 patients who had ANH (124/276) and ABT (152/276), there were no differences in (Δ) Hb, (Δ) PT, or (Δ) FIB (*P* > 0.05), while (Δ) WBC, (Δ) PLT, (Δ) APTT and (Δ) D-dimer were significantly different (*P* < 0.05). The blood transfusion method was not an independent factor for flap crisis (*P* > 0.05). The wound infection probability in patients with high post-PTs was 1.953 times greater than that in patients with low post-PTs (OR = 1.953, 95% CI: 1.232 ∼ 3.095, *P* = 0.004). A normal or overweight BMI was a protective factor for pulmonary infection, and the incidence of pulmonary infection in these patients was only 0.089 times that of patients with a low BMI (OR = 0.089, 95% CI: 0.017 ∼ 0.462). Moreover, a high ASA grade promoted the occurrence of pulmonary infection (OR = 6.373, 95% CI: 1.681 ∼ 24.163). The blood transfusion mode (B = 0.310, β = 0.360, *P* < 0.001; ANH: ln hospital stay = 2.20 ± 0.37; ABT: ln hospital stay = 2.54 ± 0.42) improved the length of hospital stay.

**Conclusion:**

Preoperative and postoperative blood transfusion (Δ) Hb, (Δ) PT, and (Δ) FIB did not differ; (Δ) WBC, (Δ) PLT, (Δ) APTT, and (Δ) D-dimer did differ. There was no difference in the effects of the two blood transfusion methods on flap crisis, incision infection or lung infection after flap reconstruction, but ANH resulted in a 3.65 day shorter average hospital stay than did ABT.

## Background

Oral and maxillofacial flap reconstruction patients often develop anemia during or after surgery due to the operation. Doctors often administer blood transfusions to patients after the loss of blood to relieve this situation. The most commonly used blood transfusion methods include allogeneic blood transfusion (ABT) and autologous transfusion. Recent research on predictable independent risk factors that are associated with postoperative complications in head and neck osseous free flap reconstruction has revealed that an age over 70 years, alcohol consumption, a history of chemotherapy and radiotherapy are associated with surgical complications [1], while another retrospective study involving 850 patients reported that intensive care unit (ICU) admission, coronary heart disease, postoperative radiotherapy surgery, and flap type are several risk factors associated with postoperative complications [2]. Michael et al. determined that the Charlson Comorbidity Index (CCI), the American Society of Anesthesiologists (ASA) classification, female sex, and inability to tolerate an oral diet were preoperative risk factors related to increased length of stay (LOS) [3]. In fact, a few specialists believe that perioperative ABT is also an important factor linked to an increased probability of flap-related complications, subsequent return to the operating room, and extension of the LOS [4, 5]. Moreover, studies have reported that the use of ABTs during cardiac or orthopedic surgeries can increase the likelihood of patients experiencing postoperative bacterial infections and may be a potential risk factor for the onset of multiple organ failure following major abdominal surgery. Indeed, several risk factors have been identified in numerous studies regarding ABT, such as infectious complications (both viral and bacterial), transfusion-related fever and allergies, hemolytic transfusion reactions associated with ABO and non-ABO antigens, acute respiratory distress syndrome (ARDS), transfusion-associated graft-versus-host disease, transfusion-associated circulatory overload, and suppression of immunomodulation. Suppression of immunomodulation is especially dangerous for individuals with malignant tumors receiving surgical treatment, leads to an increase in the recurrence rate and may increase tumor growth. ABT is likely affects mortality because of its associated complications [6, 7]. However, some studies still suggest that red blood cell transfusion (RBCT) during the preoperative period is not associated with vascular pedicle thrombosis (VPT) or the rate of microvascular free flap failure during postoperative follow-up [8]. However, the perioperative lowest Hb level and age are significant predictors of flap failure [9]. Therefore, due to the limitations of the research subjects and experimental conditions, there is still no definitive conclusions on the association between ABT and postoperative complications.

Currently, due to ongoing concerns about blood safety, there has been a growing interest in using autologous blood transfusion as a preferred substitute for allogeneic blood transfusion. Autologous transfusion is particularly effective for surgical procedures involving significant blood loss [10]. Several types of autologous transfusions have been put into clinical use: preoperative autologous blood donation (PABD), acute normovolemic hemodilution (ANH) and salvaged autotransfusion. The NHS executive has prioritized autologous transfusion techniques such as predonation, acute normovolemic hemodilution (ANH), and cell salvage as a means to decrease the necessity for allogeneic transfusion during surgery [11]. An alternative suggestion to reduce ABT in high blood loss (BL) surgery is the utilization of ANH, which has been deemed a straightforward, secure, and economical approach [12]. This process involves drawing blood from the patient just prior to surgery while simultaneously administering an appropriate amount of crystalloid or colloid fluids. This is known as ANH, which lowers the hematocrit level, thus minimizing the loss of red blood cell mass during the surgical procedure. The extracted blood is then returned to the patient’s body as autologous whole blood during or after the surgery is finished [13]. This approach may avoid most complications related to exogenous antigens in ABTs. Autologous blood is linked to fewer postoperative infections [14], a shorter hospital stay [15], and lower cancer recurrence and mortality rates than allogeneic transfusion, as it causes diminished disruption to the recipient’s humoral immune response. Nonetheless, when returned, autologous blood may not be immunologically inert, and its storage may result in elevated concentrations of signaling molecules such as cytokines and activated complement proteins, which may impact the recipient’s immune system [6]. One study showed that the use of ANH reduces the requirement and amount of ABTs in patients undergoing head and neck free flap reconstruction without increasing the incidence of postoperative complications (lung infection and length of stay were not evaluated) [16].

However, direct and effective research on whether blood transfusion can be an independent risk factor for postoperative complications and comparisons between ABT and ANH for the prognosis of flap reconstruction in oral and maxillofacial patients are still insufficient. We tested whether some common clinical features were also observed to be independent risk factors.

The aim of this research was to compare ANH with ABT in individuals who underwent oral and maxillofacial region flap reconstruction to determine whether there were any notable differences in the blood index changes or postoperative complication rates.

## Methods and materials

This single-center, retrospective, observational study was approved by the Ethics Committee of the Sun Yet-Sen Memorial Hospital, Guangzhou, China. Informed consent for this study was obtained from all subjects and/or their legal guardian(s). Data from 276 patients who required flap reconstruction via oral and maxillofacial surgery and blood transfusion were obtained at Sun Yet-Sen Memorial Hospital between September 25, 2017, and October 11, 2021, after receiving institutional ethics approval. All perioperative blood parameter measurements were performed by the same team of blood transfusion physicians and laboratory physicians. If perioperative transfusion data were unavailable, patients were excluded from the study to ensure homogeneity of the study population. The major criteria for inclusion in the study were as follows: ① aged 18 to 85 years; ② underwent elective surgery; ③ underwent flap reconstruction surgery in the oral and maxillofacial region; ④ had an intraoperative blood loss amount that exceeded 100 mL; ⑤ underwent allogeneic or autologous blood transfusion during surgery; and ⑥ had available perioperative blood parameters. The exclusion criteria were as follows: ① severe cardiopulmonary disorders, hematological disorders, liver and kidney insufficiency or abnormal preoperative coagulation function; ② contraindications to allogeneic or autologous blood transfusion; and ③ unavailable perioperative blood parameters.

## Research variables and data collection

The data were extracted from two sources: the Patient Information System of Sun Yet-Sen Memorial Hospital and the hospital blood bank database. We collected ANH and ABT patient data. To assess the impact of the two different blood transfusion techniques on postoperative complications and changes in blood indicators in patients after flap reconstruction surgery, we collected clinical characteristics, including age, sex (male or female), blood type (A, B, AB, O), BMI (≥ 18.5 vs.<18.5), anesthetic American Society of Anesthesiologists (ASA) physical status classification (I: healthy patient; II: mild systemic disease; III: severe systemic disease with functional limitation; IV: severe systemic disease with constant threat to life; V: moribund, expected survival time < 24 h), and type of disease (malignant tumor, benign tumor, osteomyelitis and osteonecrosis of the jaws), from both the ANH and ABT groups. Postoperative complications included flap crisis (the flap had a pale color, low temperature and a slow reaction of capillaries indicating arterial crisis, while silted-up and swelling indicated venous crisis), wound infection (presence of symptoms such as erythema (redness), tenderness, and discharge that was turbid (cloudy) or purulent (containing pus), with positive culture swabs), pulmonary infection (clinical symptoms such as cough, fever, chest pain, and difficulty breathing and/or radiologic abnormality requiring antibiotic treatment based on the opinion of physician), and length of hospital stay (according to a patient’s stable overall condition and good wound healing based on the comprehensive judgment of the treating clinician). Blood indicators included preoperative admission and 24-h postoperative hemoglobin concentration (Hb, g/L), leukocyte count (WBC, *10^9^/L), platelet count (PLT, *10^9^/L), prothrombin time (PT, s), activated partial thromboplastin time (APTT, s), fibrinogen (FIB, g/L), and D-dimer (D-dimer, µg/ml), and we subtracted the former from the latter to measure the changes in blood indicators, which were referred to as delta (Δ) blood indicators. Statistical analysis was employed to determine the potential associations between the two blood transfusion techniques, patient demographic characteristics, 24-h postoperative blood indicators and occurrence of postoperative complications and to determine whether there was a correlation between the two transfusion methods and changes in blood parameters.

Allogeneic blood transfusion was administered solely based on specific criteria, including (1) a hemoglobin concentration < 70 g/L (absolute indication); (2) a hemoglobin concentration < 100 g/L with signs of myocardial ischemia (relative indication); and (3) in exceptional cases, when the anesthetist determined that transfusion was necessary based on the patient’s clinical condition.

## Statistical analyses

The present study utilized the commercially available software Statistical Package for Social Science (SPSS) version 22.0 to conduct all the statistical analyses. All variables were tested for a normal distribution using the Kolmogorov‒Smirnov test. When examining and contrasting the two groups, quantitative data conforming to a normal distribution are presented as the mean ± standard deviation and were analyzed using the independent sample t test, while the median (P25, P75) and nonparametric rank sum test were utilized for comparisons between groups not satisfying a normal distribution. Qualitative data are expressed as percentages (%) and were analyzed using the chi-square test for comparisons between groups. Univariate analysis was conducted using the independent-samples t test, x^2^ test, rank sum test, variance analysis and Pearson correlation analysis at a significance threshold of *P* < 0.05. Particularly, we took the natural logarithm for the length of hospital stay to narrow the distribution difference between groups and facilitate comparison, which was represented as "ln length of hospital stay" instead in writing. Univariate analyses with statistically significant variables and those professionally considered to have an impact on outcome were included in a multivariate analysis model using logistic regression or multiple linear regression to uncover independent risk factors for postoperative complications at a test level of 0.05. To evaluate the strength of correlations, logistic regression techniques were employed to conduct a multivariate analysis on qualitative complications, with significance determined by odds ratios (ORs) accompanied by corresponding 95% confidence intervals (CIs). Similarly, multivariate analysis of quantitative complications was performed utilizing multiple linear regression techniques. Positive or negative factors were determined through the partial regression coefficient (B), and the standardized partial regression coefficient (β) was utilized to compare the intensity of each factor on outcomes. After careful consideration of both the literature review and clinical judgment, specific continuous variables were transformed into dichotomous variables. The forced entry method was employed to construct all of the regression models.

## Results

The present study involved a cohort of 256 patients who underwent head and neck flap reconstruction and met the predefined inclusion criteria between September 25, 2017, and October 11, 2021, and the study and included 124 patients who had AHNs and 152 patients who had ABTs. The patients’ preoperative clinical characteristics and postoperative complications of ANH and ABT are shown in Table 1.

**Table 1:** The patients’ clinical characteristics and postoperative complications of ANH and ABT

		ANH (*n* = 124)	ABT (*n* = 152)
Sex	Male	87 (70.2%)	112 (73.7%)
	Female	37 (29.8%)	40 (26.3%)
Age		56 (42.4, 69.6)	58.5 (45.3, 71.7)
Blood type	A	35 (28.2%)	38 (25.0%)
	B	36 (29.0%)	40 (26.3%)
	AB	3 (2.4%)	13 (8.6%)
	O	50 (40.3%)	61 (40.1%)
BMI	Underweight (< 18.5)	10 (8.1%)	16 (10.5%)
	Normal and overweight (≥ 18.5)	114 (91.9%)	136 (89.5%)
ASA-classification	I	0 (0.0%)	79 (52.0%)
	II	80 (64.5%)	73 (48.0%)
	III	41 (33.1%)	0 (0.0%)
	IV	3 (2.4%)	0 (0.0%)
Clinical diagnosis	Malignant tumor	111 (89.5%)	137 (90.1%)
	Benign tumor	9 (7.3%)	9 (5.9%)
	Osteomyelitis of jaws	4 (3.2%)	6 (3.9%)
Postoperative complications	Flap crisis	5 (4.0%)	14 (9.2%)
	Wound infection	4 (3.2%)	4 (2.6%)
	Pulmonary infection	2 (1.6%)	7 (4.6%)
	Ln length of hospital stay	2.20 (1.83, 2.57)	2.54 (2.12, 2.96)

### Univariate analysis of postoperative complications in all patients

We further explored the correlation between postoperative complications and the two blood transfusion modalities by conducting a univariate analysis of postoperative complications, including flap crisis, wound infection, pulmonary infection, and length of hospital stay, in relation to the patients’ clinical characteristics, blood transfusion modalities, and postoperative blood parameters. The outcomes of the univariate analysis of postoperative complications indicated that the occurrence of flap crisis was associated with patient age (*P* = 0.001). The incidence of wound infection was associated with the postoperative 24 h PT (*P* = 0.002). A significant correlation was observed between patient BMI (*P* = 0.043) and ASA classification (*P* = 0.016) and the incidence of pulmonary infection. The length of hospital stay was related to the blood transfusion method (*P* < 0.001), age (*P* = 0.005), patient ASA classification (*P* = 0.029), postoperative 24-h APTT (*P* = 0.026), and postoperative 24-h D-dimer (*P* = 0.042). Preliminary data analysis revealed no statistically significant associations between flap crisis, wound infection, pulmonary infection and the method of blood transfusion (*P* > 0.05). More details of the results of the univariate analysis of postoperative complications in all patients are shown in Table 2.

**Table 2:** Univariate analysis of postoperative complication in all patients

	Flap crisis		Wound infection		Pulmonary infection		Ln length of hospital stay	
	*n* = 19	*P* value	*n* = 8	*P* value	*n* = 9	*P* value	mean ± SD/*r*	*P* value
**Method of blood transfusion**		0.091		1.000		0.293		0.000
ANH(124)	5 (26.3)		4 (50.0)		2 (22.2)		2.20 ± 0.37	
ABT(152)	14 (73.7)		4 (50.0)		7 (77.8)		2.54 ± 0.42	
**Preoperative characteristics**								
Sex		0.368		0.830		0.445		0.339
Male(199)	12 (63.2)		5 (62.5)		8 (88.9)		2.40 ± 0.39	
Female(77)	7 (36.8)		3 (37.5)		1 (11.1)		2.35 ± 0.52	
Age	55.83 ± 13.30	0.001	56.28 ± 13.55	0.280	56.24 ± 13.28	0.207	0.17	0.005
	67.57 ± 11.71		61.50 ± 9.18		62.00 ± 18.01			
BMI		1.000		0.552		0.043		0.357
Underweight (< 18.5)(26)	2 (10.5)		1 (12.5)		3 (33.3)		2.31 ± 0.45	
Normal and overweight (≥ 18.5)(250)	17 (89.5)		7 (87.5)		6 (66.7)		2.40 ± 0.43	
Blood type		0.566		0.235		0.336		0.089
A(73)	4 (21.1)		5 (62.5)		1 (11.1)		2.36 ± 0.38	
B(76)	7 (36.8)		1 (12.5)		5 (55.6)		2.40 ± 0.44	
AB(16)	2 (10.5)		0 (0.0)		0 (0.0)		2.64 ± 0.48	
O(111)	6 (31.6)		2 (25.0)		3 (33.3)		2.36 ± 0.43	
ASA-classification		0.602		1.000		0.016		0.029
I(2)	0 (0.0)		0 (0.0)		0 (0.0)		2.08 ± 0.00	
II(147)	8 (42.1)		4 (50.0)		3 (33.3)		2.34 ± 0.39	
III(122)	11 (57.9)		4 (50.0)		4 (44.4)		2.44 ± 0.40	
IV(5)	0 (0.0)		0 (0.0)		2 (22.2)		2.78 ± 1.29	
Clinical diagnosis		0.811		1.000		0.347		0.627
Malignant tumor(251)	19 (100.0)		8 (100.0)		8 (88.9)		2.39 ± 0.44	
Benign tumor(15)	0 (0.0)		0 (0.0)		0 (0.0)		2.28 ± 0.33	
Osteomyelitis of jaws(10)	0 (0.0)		0 (0.0)		1 (11.1)		2.38 ± 0.29	
**Postoperative 24 h parameters (The upper rows represent the value of the parameters of the patients who did not experience the complication, the lower rows represent the value of the parameters for patients who experienced the complication)**								
Hb	111.13 ± 14.70	0.464	111.38 ± 14.87	0.641	111.20 ± 14.89	0.494	−0.011	0.853
	113.74 ± 18.17		108.88 ± 18.09		114.67 ± 17.04			
WBC	13.35 ± 4.57	0.779	13.37 ± 4.49	0.959	13.35 ± 4.46	0.747	−0.084	0.166
	13.65 ± 3.55		13.45 ± 5.04		13.85 ± 5.76			
PLT	197.43 ± 64.87	0.582	196.86 ± 64.10	0.996	196.64 ± 63.72	0.766	-0.100	0.097
	189.05 ± 48.18		196.75 ± 57.59		203.11 ± 70.41			
PT	13.09 ± 1.17	0.677	13.05 ± 1.14	0.002	13.09 ± 1.19	0.853	0.075	0.214
	12.97 ± 1.49		14.35 ± 2.00		13.01 ± 1.13			
APTT	28.88 ± 5.87	0.616	28.90 ± 6.09	0.598	28.88 ± 6.03	0.459	0.134	0.026
	29.61 ± 8.15		30.05 ± 4.08		30.40 ± 6.49			
FIB	3.67(2.81,4.63)	0.986	3.64(2.79,4.61)	0.094	3.67(2.81,4.65)	0.590	-0.042	0.487
	3.92(2.65,4.69)		4.08(3.85,4.91)		4.15(2.49,4.94)			
D-dimer	1.98(1.14,3.27)	0.193	2.02(1.15,3.35)	0.833	1.99(1.18,3.28)	0.867	0.122	0.042
	2.90(1.35,5.27)		1.81(1.09,3.06)		2.14(0.89,6.34)			

#### Flap crisis

The results of the univariate analysis suggested that the occurrence of flap crisis was not associated with the blood transfusion method (*P* > 0.05) but was related to patient age. Subsequently, the blood transfusion method, as a possible factor influencing flap crisis, was also included in the binary logistic regression analysis, and the results showed that neither the method of blood transfusion (OR = 2.307, 95% CI: 0.804 ∼ 6.624, *P* = 0.120) nor age (OR = 1.028, 95% CI: 0.988 ∼ 1.069, *P* = 0.171) was an independent risk factor for the development of flap crisis, as shown in Table 3.

**Table 3:** Independent risk factors for flap crisis

	Flap crisis	No flap crisis	OR, 95% CI	*P* value
**Method of blood transfusion**			2.037 (0.804–6.624)	0.120
ANH	5	119		
ABT	14	138		
**Preoperative characteristics**				
Age	67.57 ± 11.71	55.83 ± 13.30	1.028 (0.988–1.069)	0.171

#### Wound infection

The results of the univariate analysis suggested that wound infection was associated with postoperative 24-h PT (*P* = 0.002); therefore, based on the binary logistic regression analysis, postoperative 24-h PT (OR = 1.953, 95% CI: 1.232 ∼ 3.095, *P* = 0.004) was an independent risk factor for the occurrence of wound infection, and patients with high postoperative 24-h PT had a 95.3% greater chance of developing wound infection than those with low postoperative 24-h PT, as shown in Table 4.

**Table 4:** Independent risk factors for wound infection

	Wound infection	No wound infection	OR, 95% CI	*P* value
**Postoperative 24 h parameters**				
Postoperative 24 h PT	14.35 ± 2.00	13.05 ± 1.14	1.953 (1.232,3.095)	0.004

#### Pulmonary infection

The manifestation of pulmonary infection was correlated with patient BMI (*P* = 0.043) and ASA classification (*P* = 0.016); therefore, further binary logistic regression analysis revealed that compared with underweight BMI patients, normal and overweight BMI patients had a 91.1% reduced incidence of pulmonary infection (OR = 0.089, 95% CI: 0.017 ∼ 0.462). Moreover, a high ASA grade was shown to promote the occurrence of pulmonary infection (OR = 6.373, 95% CI: 1.681 ∼ 24.163), as shown in Table 5.

**Table 5:** Independent risk factors for pulmonary infection

	Pulmonary infection	No pulmonary infection	OR, 95% CI	*P* value
**Preoperative characteristics**				
BMI			0.089 (0.017, 0.462)	0.004
Underweight (< 18.5)	3	23		
Normal and overweight (18.5)	6	244		
ASA-classification			6.373 (1.681, 24.163)	0.006
I	0	2		
II	3	144		
III	4	118		
IV	2	3		

#### Length of hospital stay

Based on the findings in the univariate analysis conducted on qualitative and quantitative data, it can be inferred that the length of hospital stay was associated with the blood transfusion method (*P* < 0.001), age (*P* = 0.005), patient ASA classification (*P* = 0.029), postoperative 24 h APTT (*P* = 0.026), and postoperative 24 h D-dimer (*P* = 0.042). Therefore, we further conducted a multiple linear regression analysis involving the above relevant factors to explore the independent risk factors for the length of hospital stay. The method of blood transfusion (B = 0.310, SEx = 0.076, β = 0.360, *P* < 0.001; ANH: ln length of hospital stay = 2.20 ± 0.37; ABT: ln length of hospital stay = 2.54 ± 0.42) had a positive impact on the length of hospital stay among the indices we observed, because the length of hospital stay in the ANH group was 6.23 ∼ 13.07 days, while in the ABT group, it was 8.33 ∼ 19.30 days after conversion. The results are shown in Table 6.

**Table 6:** The independent risk factors for length of hospital stay

	Nonstandardized coefficient	Standard error	Standardized coefficient	t	*P*	Collinear statistics
	B		β			VIF
(constant)	1.472	0.180	/	8.157	0.000	/
Method of blood transfusion	0.31	0.076	0.36	4.098	0.000	2.518
ASA-classification	0.051	0.048	0.065	1.055	0.293	1.236
Age	0.003	0.002	0.105	1.665	0.097	1.289
Postoperative 24 h APTT	0.004	0.004	0.055	0.967	0.334	1.066
Postoperative 24 h D-dimer	0.004	0.007	0.028	0.485	0.628	1.112

### Variation in hematological variables of ANH patients and ABTs

We aimed to explain the possible mechanisms underlying the influence of different transfusion methods on postoperative complications from the perspective of hemochanges. We explored the correlation between the change in blood parameters (Δ blood indices), referring to the difference between the postoperative 24-h blood tests and preoperative blood tests, and the two blood transfusion modalities by conducting a group comparison. We found that the ΔHb, ΔPT and ΔFIB of the ANH and ABT groups were not significantly different (*P* > 0.05). Moreover, the results suggested that the variation in hematological variables, including ΔWBC, ΔPLT, ΔAPT, and ΔD-dimer, differed between the ANH and ABT groups, as shown in Table 7.

**Table 7:** Univariate analysis of variation in hematological variables between patients with ANH and ABT

	ANH	ABT	t/F	*P* value
**Variation in blood parameters**				
Δ Hb	−24.50(−31.75, −16.00)	−20.00(−29.75, −14.25)	−1.766	0.077
Δ WBC	7.45 ± 4.37	5.55 ± 3.82	3.846	0.000
Δ PLT	−54.50(−80.00, −28.00)	−64.50(−103.25, −37.25)	−2.499	0.012
Δ PT	1.20(0.60,1.80)	1.30(0.63,1.90)	−0.32	0.749
Δ APTT	1.35(−1.20,4.13)	2.60(0.03,6.65)	−3.135	0.002
Δ FIB	0.16(−0.52,0.86)	−0.02(−0.73,0.89)	−0.703	0.482
Δ D-dimer	1.41(0.63,2.36)	1.82(0.89,3.41)	−2.323	0.020

## Discussion

Radical tumor resection and flap reconstruction are generally acknowledged as procedures with a high level of risk. This is attributable, in part, to the intricate anatomical structures and extensive vascular distribution within the surgical site, which confer an elevated risk of significant blood loss. Therefore, patients who undergo flap reconstruction surgery may be appropriate candidates for ANH. In parallel, postoperative complications of flap reconstruction are also partially related to changes in blood parameters caused by blood transfusion. To date, there have been few studies on the effects of these two transfusion methods on oral and maxillofacial flap reconstruction surgery. Our study was designed to compare the impact of two blood transfusion methods on blood index changes and postoperative complication rates after oral and maxillofacial flap reconstruction surgery. Despite the lack of blinding of the anesthesiologist responsible for determining the necessity of blood transfusion, which determined the patients’ group assignment, the criteria for transfusion indication were standardized in our study. According to the results of the variation in hematological indices for ANH and ABT, we found that the ΔWBC, ΔPLT, ΔAPPT and ΔD-dimer between the ANH and ABT groups were significantly different, while the ΔHb, ΔPT, and ΔFIB showed no differences. In our study, the ΔWBC of the ANH (7.45 ± 4.37) was significantly greater than that of the ABT (5.55 ± 3.82), indicating that ANH could recruit more white blood cells than ABT. Although the increase in the ΔWBC is partly due to the inflammatory state caused by surgery, biochemical evidence suggests that infusion of stored older red blood cells can lead to lysis and an inflammatory state, which resulted in an increase in the ΔWBC in the ABT group [17]. However, we believe that the reason that the ANH group had a higher increase in their white blood cells may be because the diluted blood led to less loss of white blood cells, and fresh white blood cells were infused during surgery, which means that more autologous white blood cells of the patients were preserved.

In our study, the difference between the preoperative PLT and postoperative PLT of the two transfusion methods was statistically significant, and the platelet count exhibited variability, which can be explained by the combined impact of the reintroduction of functional platelets and the infusion of autologous blood after the surgery, and this result was similar to that of previous studies [10]. Moreover, the results were in agreement with those of previous studies [11, 18], which did not reveal any notable impact of ANH on the platelet count. Similar results were obtained in a retrospective study by Naveen Bansal et al., who indicated that storing autologous blood at room temperature maintained platelet function, enabling the prompt availability of platelets and clotting factors immediately after the surgical procedure [10]. Both of these factors might prevent a decrease in the PLT levels in patients undergoing ANH.

Our study yielded comparable outcomes: the Δ APTT and Δ D-dimer of the two transfusion methods were significantly different.

Δ APTT and Δ PT are blood indices related to blood coagulation. It is the difference between the value after blood transfusion and the value before blood transfusion. This reflects an increase in blood coagulation time, that is, a decrease in blood coagulation ability. The greater the difference, the more obvious the decrease in blood coagulation ability. Our data showed that the difference between the ANH and ABT was not the same; statistically, the Δ APTT of the ANH was lower than that of the ABT, but neither of the Δ PT values were significantly different. We believe that because APTT reflects endogenous coagulation ability, all coagulation factors involved in endogenous coagulation exist in the blood, starting from vascular endothelial injury and intravascular coagulation, and the Δ APTT of patients undergoing ANH is lower than that of patients undergoing ABT, suggesting that the decrease in blood coagulation ability of ANH is smaller than that of ABT, which may be related to the retention of more endogenous coagulation factors in ANH. PT reflects the ability of exogenous coagulation. Exogenous coagulation is initiated by the exposure of damaged tissue to collagen fiber-activating tissue factor (factor III), which is then transmitted to other downstream coagulation factors. We hypothesize that ANH cannot activate more tissue factors than ABT, so the Δ PT showed no significant difference. Endogenous coagulation and exogenous coagulation occur at the same time after flap reconstruction and vascular anastomosis. In general, the blood coagulation potential of ANH is greater, which has a positive effect on preventing continuous bleeding after flap reconstruction in clinical practice and preventing postoperative anemia caused by a continuous reduction in Hb levels. FIB is coagulation factor I, which is converted into fibrin monomers under the action of thrombin and is the main protein involved in the process of coagulation. No significant difference in ΔFIB was detected, indicating that the degradation of FIB in stored allogeneic blood was not obvious. D-dimer is a specific degradation product produced by fibrin monomers cross-linked by activation factor XIII and then hydrolyzed by fibrinolytic enzymes. It is a specific fibrinolytic process marker that mainly reflects fibrinolytic function. As long as there is activated thrombosis and fibrinolytic activity in the body’s blood vessels, D-dimer levels will increase. The statistical results showed that the ΔD-dimer of the ANH group was lower than that of the ABT group, which can be explained by the lower D-dimer level increase caused by ANH, and this means that ANH leads to less intravascular activated thrombus formation after the operation. Thrombosis is an important inducer of flap crisis. Therefore, ANH may be superior to ABT in reducing the occurrence of flap crisis. Research has shown that long-term storage of red blood cells results in increased levels of unbound hemoglobin (Hb) and iron in the supernatant released from hemolyzed cells, causing a reduction in oxygen-carrying capacity, but total Hb is not greatly affected [19].

### Flap crisis

In our study, we did not observe a statistically significant difference (*P* = 0.091) in the occurrence of flap crisis between the ANH (*n* = 5 in 124) and ABT groups (*n* = 14 in 152) (Table 2), nor did we observe that the method of transfusion (*P* = 0.120) or patient age (*P* = 0.171) were independent risk factors for flap crisis (Table 3), even though the odds ratios reached 2.037 (0.804, 6.624) and 1.028 (0.988, 1.069), respectively, as shown in Table 3. However, there are many reasons for the occurrence of flap crisis after flap reconstruction surgery, and in a study of 1072 cases of flap crisis after flap surgery, it was mentioned that the causes of flap crisis were venous thrombosis, vascular obstruction, arterial occlusion, compression of the perforator by muscle and mandible, twisting of the pedicle, internal jugular vein thrombosis, and injury to the perforator noted during the operation [20]. In our study, which included a multivariate analysis of cases in which flap crisis occurred after flap surgery, we found that the occurrence of flap crisis was not associated with blood transfusion methods (*P* = 0.120) or patient age (*P* = 0.171). In fact, in some retrospective studies involving ABT or ANH alone, the multifactorial nature of flap survival makes the results difficult to interpret, leading to a variety of conclusions on the impact of different blood transfusion methods on flap crisis. Kim et al. [9]. reported that perioperative ABT did not increase the risk of flap crisis, and the lowest Hb level and age during the perioperative period were important predictive factors for flap crisis. Similarly, Torres Fuentes et al. [8]. and Puram et al. [21]. argued that allogeneic red blood cell infusion was not associated with the flap survival rate or vascular pedicle thrombosis. Moreover, the results of the association between ANH and flap crisis have not been consistent. A retrospective study conducted by Hill et al. [22]. found a significant correlation between a low hematocrit value (below 30%) and free flap failure. However, in a large retrospective study one year later, Mlodinow et al. [23]. reported that anemia caused by a low hematocrit value was not associated with free flap failure in free flap reconstruction surgery. Furthermore, in animal experiments with adult minipigs and rats, ANH has been proposed to improve the oxygenation and blood perfusion of ischemic and hypoxic flap tissue, thereby increasing the flap survival rate [24, 25]. Although there were some limitations in the above research and only a single blood transfusion method was studied at one time, it was not difficult to determine whether the two blood transfusion methods had distinct effects on the occurrence of flap crisis because of their differential changes in Hb and adequate blood perfusion of free flaps. In our study, we did not observe significant differences in the ΔHb between the ABT group and the ANH group (ABT=-20.00 (-29.75, 14.25), ANH=-24.50 (-31.75, 16.00), *P* = 0.077). A systematic review on blood perfusion showed that ANH could lead to a decrease in systemic HCT and blood viscosity. At the same time, due to the smaller decrease in average capillary HCT compared to systemic HCT and the well-distributed red blood cells, the oxygenation and blood perfusion of the free flaps would also be improved [26]. However, ANH was more likely to cause hypercoagulable blood than was ABT, thereby increasing the risk of flap crisis. There is literature indicating that blood dilution could lead to an enhanced coagulation state [27]. Further studies have shown that lower blood viscosity and changes in rheology induce initial hemostasis [28], changes in laminar flow, and an increase in turbulence, which increase the risk of thrombosis [29]. Therefore, based on the above factors, we speculated that the combination of ΔHb without significant difference and the promoting and inhibiting factors of blood perfusion, which explains why there was no difference in the occurrence of flap crisis between the two blood transfusion methods.

### Wound infection

In our study, wound infections (WI) occurred in the ANH group (*n* = 4) and in the ABT group (*n* = 4), but the difference in the frequency of WI between the two groups was not statistically significant. Wound infection was defined as the presence of symptoms such as erythema (redness), tenderness, and discharge that were turbid (cloudy) or purulent (containing pus), with positive culture swabs. The requirement for blood transfusion also indicated the possibility of extensive tissue ablation and a sizable surgical defect that would necessitate reconstruction, resembling the risks associated with postoperative WI in cases where free flap or PMMCF reconstruction is needed to access the upper aerodigestive tract or address significant surgical defects [30]. The transfusion of ANH was found to trigger complement system activation and elevated plasma levels of IL-6 in healthy volunteers over a 7-day period in a previous study, potentially contributing to a decrease in the postoperative infection risk [6]. An additional investigation involving 1,000 patients with oral cavity squamous cell carcinoma revealed that the rate of postoperative WI was 19.8%, and perioperative blood transfusion emerged as an independent factor that was significantly associated with the occurrence of postoperative WI [31]. In our study, the results of the univariate analysis and subsequent multivariate analysis showed that the 24-h postoperative PT was an independent risk factor for WI (*P* = 0.004) and demonstrated that based on its odds ratio, 24-h postoperative PT was a risk factor for WI occurrence (OR = 1.953, 95% CI= (1.232, 3.095)). No significant difference was noted in our study between WI and the method of transfusion or platelet count (*P* > 0.05). In contrast, Dean Fergusson et al. reported that ABT was believed to induce an immunosuppressive effect that heightened the risk of postoperative WI [32]. Pelczar et al. reported that a high platelet count was significantly associated with WI [33]. Studies by Sidharth V. Puram et al. have shown that perioperative transfusion of free flaps increases wound dehiscence rates and that the rates of fistula formation and wound infection tend to be greater in transfused patients than in nontransfused patients. One animal experiment showed that blood transfusions trigger the release of inflammatory cytokines, such as interleukin-8, which impair wound healing [34], although the basis for this difference is debatable.

### Pulmonary infection

In our study, wound infection occurred in the ANH group (*n* = 2) and in the ABT group (*n* = 7), but the difference in the frequency of wound infections between the two groups was not statistically significant. Our study suggested that there was no statistically significant difference between the two transfusion modalities and the occurrence of postoperative pulmonary infections (*P* > 0.05), and our study revealed that the preoperative BMI (*P* = 0.004) and ASA classification (*P* = 0.006) were independent risk factors for pulmonary infection. These results suggested that underweight (BMI < 18.5) patients (3/26) were more likely to develop postoperative pulmonary infection than were normal and overweight (BMI ≥ 18.5) patients (6/250), while the incidence of postoperative pulmonary infection increased as the ASA classification increased. Moreover, their odds ratio and 95% CI reached 0.089 (0.017, 0.462) for BMI and 6.373 (1.681, 24.163) for ASA classification, which indicated that BMI acted as a protective factor and that ASA classification was a highly correlated risk factor. However, unlike our study, a retrospective study by Sidharth V et al. revealed that there was a significant association between pulmonary infection and ABT [21], but the specific reasons for this association need further exploration.

### Length of hospital stay

The length of hospital stay was a comprehensive indicator that was determined by multiple factors. Patients could only be discharged when their overall condition was stable; thus, the length of hospital stay to some extent reflected the success of surgery, with fewer postoperative complications and a faster recovery speed. In this study, we took the natural logarithm for the length of hospital stay to narrow the distribution difference between groups and facilitate comparison. We found that the method of blood transfusion, patient age, preoperative ASA classification, 24-h postoperative APTT and D-dimer level were associated with the length of hospital stay according to univariate analysis (*P* < 0.05). Similar to the conclusions drawn from Clark JR et al.‘s study, hospitalization duration is prolonged by several factors, including preoperative hemoglobin levels below 11 g/dl, ASA classification, active smoking within two weeks prior to surgery, the presence of a tracheotomy, and a crystalloid infusion rate exceeding 130 mL/kg/24 h [35]. However, multiple linear regression analysis revealed that only the blood transfusion method was an independent risk factor for length of hospital stay (*P* < 0.001), with the standardized partial regression coefficient (β) reaching 0.360, which is far greater than that of other factors. The mean ln length of stay was significantly different between the ANH group (2.20 ± 0.37) and the ABT group (2.54 ± 0.42). Compared to the ABT group, the ANH group had an average reduction of 3.65 days, with a maximum reduction of 13.06 days in the length of hospital stay. This outcome corroborated the conclusion of Bennett et al., who reported that patients who underwent ANH had shorter hospital stays than did patients in the control group. Nevertheless, Oishi et al. reported no significant difference in the length of hospital stay between the ANH group and the control group [18]. It can be concluded that mostly using ANH rather than ABT is a key measure for reducing the length of hospital stay and improving the quality of patient survival.

After doing an analysis of the postoperative blood parameter changes, our results suggested that the changes in the ΔWBC and ΔPLT were statistically significant, and our previous results revealed that wound infection in the ABT group and ANH group was statistically significant; moreover, the changes in these indicators may be associated with postoperative wound infection, which is consistent with the results of Sidharth V et al. [21].

The study also has limitations. The limitations of the study include its restricted external validity because this study was conducted at a single institution and focused solely on patients admitted for potential treatment. Additionally, as a retrospective study rather than a cohort study, it is challenging to establish causality. Furthermore, despite standardized treatment protocols at our institution, unavoidable individual variances among surgeons may impact the results.

## Conclusion

In this study, a statistical analysis of 124 patients in the ANH group and 152 patients in the ABT group revealed that there was no difference in the preoperative or postoperative ΔHb, ΔPT or ΔFIB between the ANH and ABT groups, but there was a difference in the ΔWBC, ΔPLT, ΔAPTT and ΔD-dimer. There was no difference in the effect of the two transfusion methods on flap crisis, incisional infection or pulmonary infection after flap surgery. In particular, the average length of hospital stay was 3.65 days shorter in the ANH group than in the ABT group. Therefore, in the face of adverse effects and unpredictable complications of allogeneic transfusion and limited clinical blood resources, ANH is a superior transfusion method to ABT.

## Data Availability

The datasets generated and analyzed during the current study are not publicly available (ownership of data) but are available from the corresponding author upon reasonable request.
